# In vitro regeneration of Ugandan passion fruit cultivars from leaf discs

**DOI:** 10.1186/s13104-019-4469-8

**Published:** 2019-07-16

**Authors:** Samuel Tuhaise, Jesca L. Nakavuma, John Adriko, Kenneth Ssekatawa, Andrew Kiggundu

**Affiliations:** 1 National Agricultural Biotechnology Centre, National Agricultural Research Laboratories, Kawanda-NARO, P.O Box 7065, Kampala, Uganda; 2 College of Veterinary Medicine, Animal Resources and Biosecurity, Makerere University, P. O. Box 7062, Kampala, Uganda; 3 Donald Danforth Plant Science Center, St. Louis, MO USA; 4 Department of Biochemistry, Faculty of Biomedical Sciences, Kampala International University-Western Campus, P. O. Box 71, Bushenyi, Uganda

**Keywords:** *Passiflora edulis*, Shoot Induction Media, Regeneration system, Gibberellic acid, α-Naphthaleneacetic acid, 6-Benzylaminopurine, Organogenesis

## Abstract

**Objective:**

Passion fruit improvement efforts by conventional breeding have had limited success calling for research into alternative approaches such as tissue culture and genetic engineering. An efficient and reproducible regeneration system is a prerequisite for successful genetic engineering. Currently, there is no reliable regeneration system for Uganda’s passion fruit varieties owing to the high heterogeneity of the *Passiflora* genus. Therefore, this study aimed at establishing an efficient and reproducible regeneration system for Uganda’s *Passiflora edulis* f. *flavicarpa* (yellow passion fruit) and *Passiflora edulis* f. *edulis* (purple passion fruit) for routine utilization with an ultimate goal of improving its agronomic value.

**Results:**

The study successfully induced shoots by both direct and indirect organogenesis for the yellow passion fruit variety. Highest shoot induction frequency (14.85%) was achieved on 8.9 μM BAP while 7.9 μM BAP did not initiate any shoots. Optimal shoot elongation and rooting was achieved on 0.44 μM BAP and 5.37 µM α-naphthaleneacetic (NAA) respectively. Rooted yellow passion fruit plantlets were successfully weaned with over 65% survival rates. It took approximately 6 months to produce a weaned healthy passion fruit plant. The purple passion fruit variety proved to be recalcitrant to tissue culture with no successful shoot or callus induction.

**Electronic supplementary material:**

The online version of this article (10.1186/s13104-019-4469-8) contains supplementary material, which is available to authorized users.

## Introduction

Horticulture is one of the fastest growing sectors in Uganda exporting products worth US$100 million per year mainly to the European Union. Passion fruit (*Passiflora edulis*) growing and export is an important contributor to the horticultural sector employing over a million farmers. Uganda annually earns over US$ 200,000 from passion fruit exports [1, 2]. In addition to its economic contribution, passion fruit is also valued for its medicinal, nutritional and ornamental roles [2–4].

However, there are several constraints to passion fruit productivity especially viral diseases, pests and environmental stress [5]. Declining yields from passion fruit farming due to the above constraints have initiated widespread enterprise abandonment by farmers [5, 6]. Conventional crop improvement programmes to develop resistant varieties to some of these problems are currently ongoing but with limited success mainly due to reproductive barriers, failures in interspecific crosses, low recovery of hybrids and the process being slow [7–10]. This has necessitated for research into other alternative approaches to compliment traditional breeding endeavors for passion fruit improvement such as plant tissue culture and genetic engineering [10]. However, an efficient and reproducible regeneration system is a prerequisite for successful genetic engineering.

*Passiflora* is highly diverse with approximately 520 species; this high heterogeneity of the genus makes the application of tissue culture technology very difficult due to various physiological and developmental problems [11, 12]. Due to this high genetic diversity among species of *Passiflora*, there was need to optimize existing regeneration systems to suit Uganda’s *Passiflora* cultivars. This study therefore aimed at establishing an efficient and reproducible regeneration system for Uganda’s *Passiflora edulis* f. *flavicarpa* (yellow passion fruit) and *Passiflora edulis* f. *edulis* (purple passion fruit) for routine utilization with an ultimate goal of improving its agronomic value.

## Main text

### Materials and methods

#### Study site and plant material

The study was conducted at the Biotechnology Centre housed at the National Agricultural Research Laboratories (NARL), Kawanda. The yellow passion fruit variety was chosen for the study because its widely cultivated, tolerant to diseases, larger fruit quality and vigorous vine while the purple variety was considered for its strong aroma, flavour and taste [13–15]. High quality passion fruits plants were purposively sampled from the Kawanda nursery according to parameters like yield, stature, growth rate and vigour. These plants were used to start a basin explant mother garden in an insect proof screen house to provide continuous supply of leaves for regeneration studies. Explant preparation and direct shoot organogenesis of the yellow and purple passion fruit varieties was done following the protocol described by [16] with a few modifications.

#### Preparation of explant materials for culturing

Fresh young leaves were rinsed with 1% (*v/v*) detergent for 5 min to remove adherent particles. Leaves were soaked for 25 min in 2% (*v/v*) fungicide (GOLDAZIM 500 SC; *Active Ingredient*; Carbendazim 500 g L^−1^), surface sterilized with 70% (*v/v*) ethanol for 2 min and finally disinfected in 2.5% sodium hypochlorite augmented with two drops of TWEEN-20 for 10 min with occasional agitation [16]. Leaves were rinsed five times in sterile distilled water to wash them free of the previously used solution under aseptic conditions after each surface sterilization step. Leaf discs (± 1 cm^2^) were cultured onto MS medium [17] media supplemented with varying benzylaminopurine (BAP) concentrations to induce shoot development. Leaf discs were cultured with their adaxial side in contact with the media.

#### Screening different BAP concentrations for shoot induction

Four different BAP concentrations at 6.9 µM (1.6 mg L^−1^), 7.9 µM (1.8 mg L^−1^), 8.9 µM (2 mg L^−1^) and 9.9 μM (2.2 mg L^−1^) were tested for adventitious shoot induction from leaf discs. Shoot Induction Media (SIM) was prepared by dissolving full strength MS basal salts and vitamins, sucrose (30 g L^−1^), appropriate BAP concentration and pH adjusted to 5.8. Gelrite (2.4 g L^−1^) was added to SIM prior to autoclaving at 121 °C for 15 min. All biochemicals and media constituents used were procured from Duchefa Biochmie (RV Haarlem, Netherlands). Each BAP concentration had 10 replicates each with six explants (leaf discs), totaling 60 explants per treatment. The plates were kept in the dark (8/16 h light/dark cycle) for a month after which they were transferred to light (16/8 h light/dark cycle) at 26 ± 2 °C.

#### Shoot elongation

Successfully induced shoots were transferred to shoot elongation media supplemented with either 2.9 μM GA_3_ or 0.44 μM BAP. Multiple induced shoots were carefully separated using a scalpel and cultured on MS supplemented with 0.44 μM BAP to promote further shoot proliferation and elongation.

#### Root induction, acclimatization and plant development

Root induction of elongated passion fruit leafy shoots (at least 3 cm) was done on MS supplemented with either 5.37 µM (1 mg L^−1^) NAA or 10.74 µM (2 mg L^−1^) NAA. Successfully rooted plantlets were washed to remove adhered agar and then gently placed in disposable plastic cups containing sterile soil mixture in the ratios 3:2:1 (loam, manure, sand). Plantlets were covered with a clear disposable cup to maintain high humidity. Plants that developed new leaves were transferred to pots where they developed normally with regular watering. Regeneration data was analyzed using GraphPad Prism 7.01.

### Results

#### Screening different BAP concentrations for shoot induction

There was successful shoot induction from yellow passion fruit leaf discs however no shoots were induced for the purple variety. A total of twenty yellow passion fruit shoots were induced from all the four BAP concentrations tested for both replicates (Table 1). Shoots were induced via both direct and indirect organogenesis, a total of 18 shoots (81.82%) were induced directly from leaf discs while 4 shoots (18.18%) were induced via an intervening callus phase. Media supplemented with 8.9 μM BAP showed the highest shoot induction frequency producing thirteen shoots translating in 65% of the total shoots initiated while no shoots were induced on 7.9 μM BAP (Table 1). The highest average shoot initiation frequency of 14.85% (15/101 × 100) was recorded on 8.9 μM BAP.

**Table 1:** Effect of different BAP concentrations on shoot organogenesis from yellow passion fruit leaf discs

Treatment (n = 60; *shoots per replica for each treatment)*	Number of infected explants	Number of sprouts	Time to first shoot in weeks	No. of plantlets	Plantlet regeneration %	Average Reg. %
6.9 µM BAP						
Replicate 1	10	4	7	2	4	3.05
Replicate 2	12	4	9	1	2.1	
7.9 µM BAP						
Replicate 1	8	0	–	0	0	0
Replicate 2	6	0	–	0	0	
8.9 µM BAP						
Replicate 1	8	10	11	6	11.5	14.85
Replicate 2	11	13	10	9	18.2	
9.9 µM BAP						
Replicate 1	11	5	14	2	4.1	3.95
Replicate 2	8	7	13	2	3.8	

Shoot regeneration % = [Induced shoots from leaf discs/(Number of leaf discs initiated-Infected explants) × 100]. For each *replicate*, a total of 60 leaf discs were initiated; *infected explants* were those that were lost due to either bacterial or fungal contamination during culturing on SIM

**Fig. 1:**
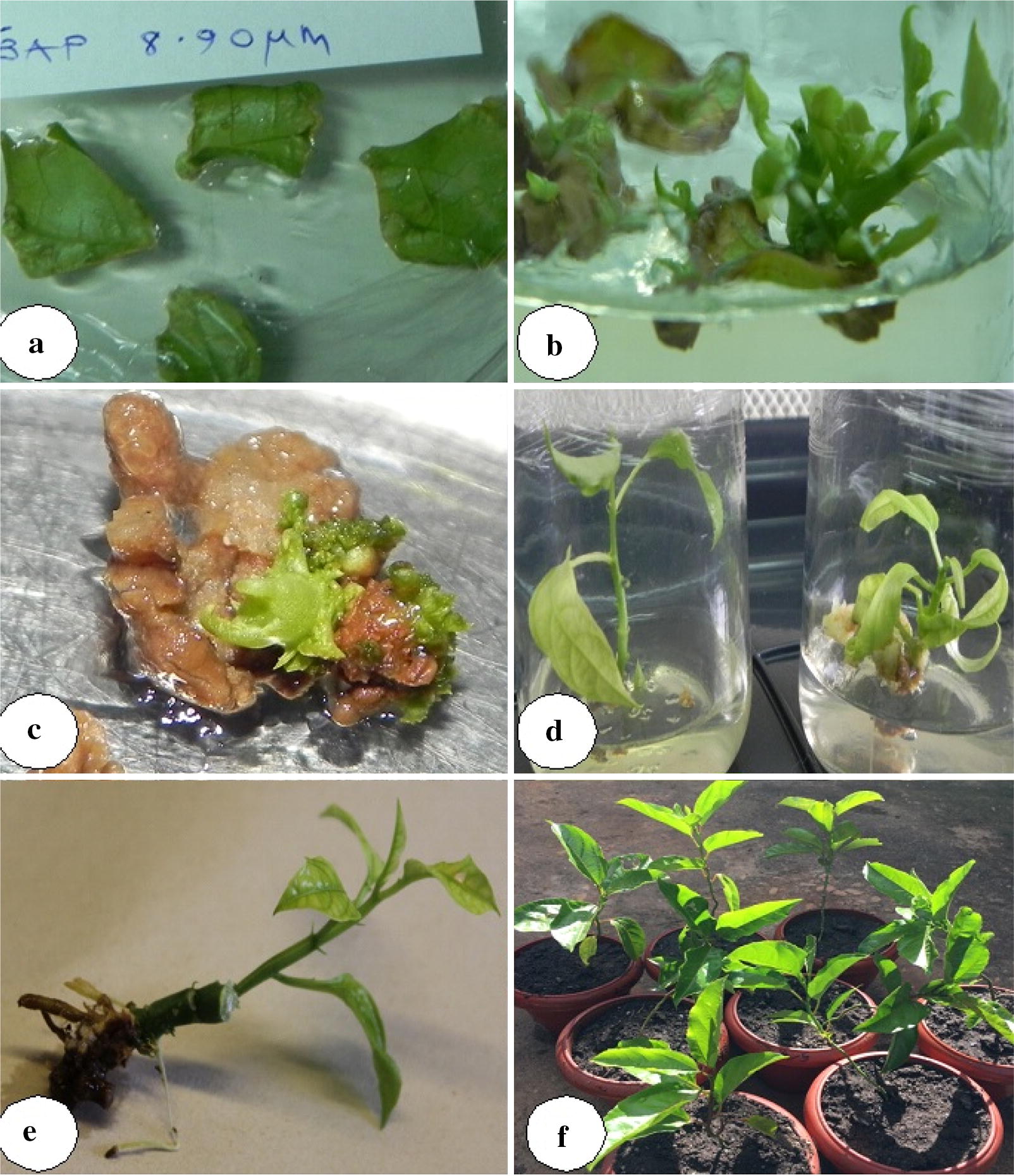
Direct shoot organogenesis of yellow passion fruit; **a** 2½ week old leaf discs on 8.9 µM BAP; **b** direct shoots and buds originating from a 2½ month old leaf discs; **c** direct shoot bud induction from a 3 month necrotic leaf disc on 9.9 µM BAP; **d** elongated shoots after a month on 0.44 µM BAP; **e** adventitious roots induced on 5.37 µM NAA after 3 weeks; **f** successfully weaned yellow passion fruit plants in the screen house

On average, a single shoot was induced from 20 initiated leaf discs. However, there was observed multiple shoot induction with one leaf disc cultured on 8.90 μM BAP giving off three distinct shoots. Shoots generally sprouted out along the injured edges of the leaf disc (Fig. 1b).

**Fig. 2:**
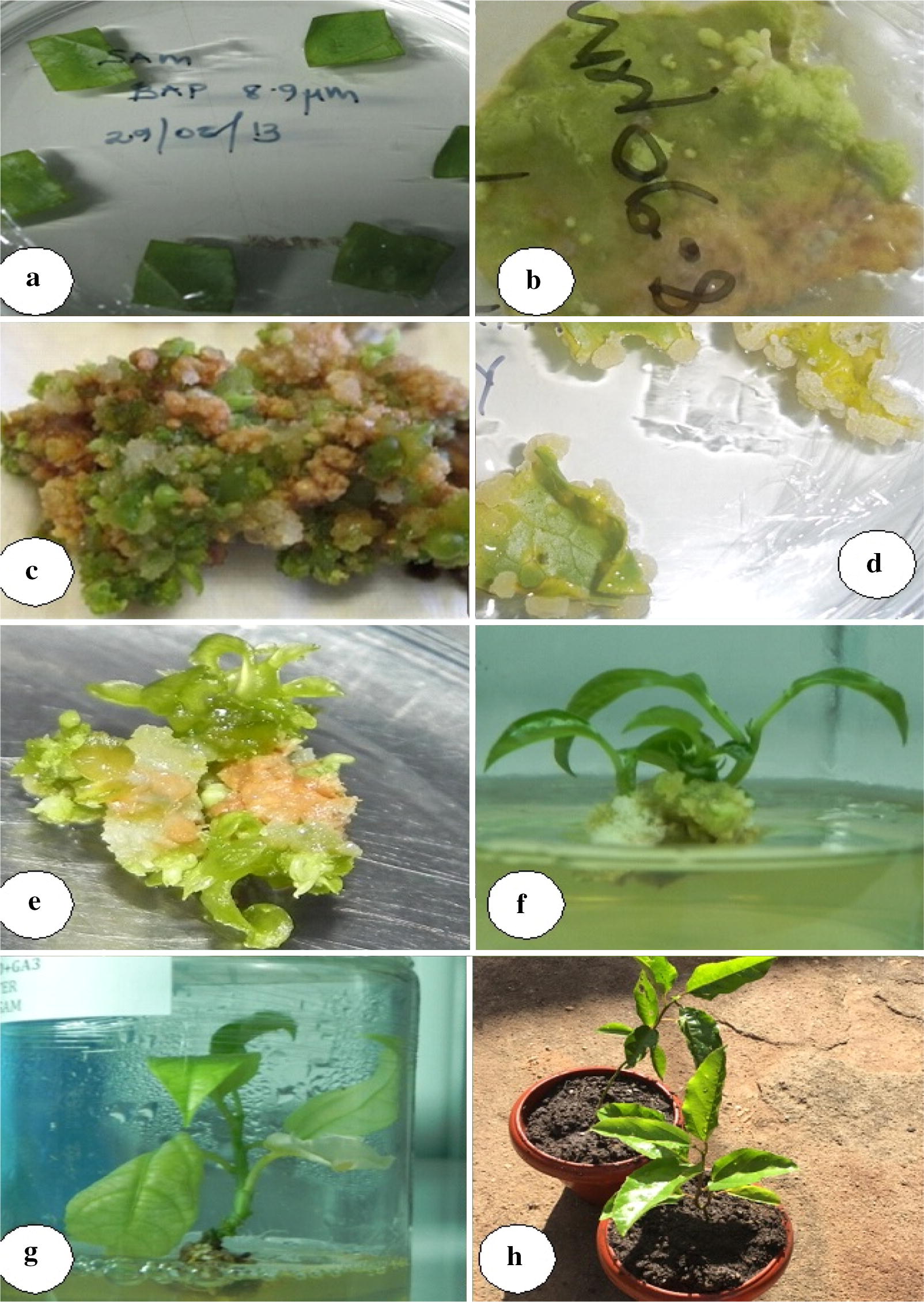
Indirect shoot regeneration of yellow passion fruit via the callus phase; **a** 2 week old leaf discs on 8.9 µM BAP; **b** callus starting to grow from a leaf disc on 9.9 μM BAP; **c** embryogenic callus with protruding buds developing on a leaf disc after 2½ months; **d** non embryogenic callus growing from injured edges of leaf discs; **e** indirect organogenesis of sprouts from embryogenic callus; **f** young shoots sprouting out of 3½ month old embryogenic callus; **g** elongated leafy shoot on 0.44 µM BAP; **h** successfully weaned yellow passion fruit plants

None of the tested BAP concentrations successfully induced any shoots, buds or callus from purple passion fruit leaf discs. All four BAP concentrations did not stimulate cell division on purple passion fruit leaf discs. Leaf discs instead browned and became necrotic after 12 weeks. Approximately a quarter of all initiated discs gave off callus from their injured portions (Fig. 2). The calli grew large with subsequent sub-culturing producing large nodular mass which differentiated into embryogenic and non-embryogenic portions. Embryogenic sectors were compact, highly nodular green portions while the non-embryogenic regions were friable loose nodular brown portions. The embryogenic callus induced numerous sprout buds (Fig. 2c). Some of these buds developed into shoots on prolonged culture of about 8 weeks.

#### Shoot elongation

Most of the induced shoots did not grow to the required shoot length for successful rooting and were cultured on elongation media with varying results (Additional file 1: Table S1). Shoots cultured on media supplemented with 2.9 μM GA_3_ showed minimal increase in shoot height after 6 weeks. However, shoots grown on 0.44 μM BAP showed an increase in shoot length of about 2–3 cm after 4 weeks (Fig. 1f). This media composition (0.44 μM BAP) sometimes resulted in induction of sprout buds from the base of the original cultured plantlet after a month (Fig. 1e). These off shoots grew normally attaining the required height for rooting. This phenomenon increased the total number of elongated shoots ready for rooting from the original 22 induced from leaf discs to a total of 34 elongated shoots.

#### Root induction, acclimatization and plant development

There was successful rooting of approximately 60% of all elongated shoots cultured on NAA with an average of three adventitious roots (Additional file 1: Table S2). Shoots cultured 5.37 µM NAA induced thin long adventitious roots at the base of the stem after 4 weeks with minimal basal callus production. However, shoots cultured on 10.74 µM NAA induced thick short roots after 3½ weeks but with extensive non-embryogenic nodular friable callus at the shoot base. Successful regeneration of a healthy rooted in vitro plantlet took approximately 5 months from leaf disc induction via direct organogenesis. Weaning of in vitro rooted plants showed over 65% survival rate in the screen house. It took approximately 6 months to obtain weaned plants via direct organogenesis compared to approximately 7 months via the indirect route.

### Discussion

Plant regeneration remains an important bottleneck for successful establishment of highly reproducible transformation systems [18, 19]. The study successfully induced shoots for the yellow variety. Successful shoot induction was probably due to the BAP concentrations used. The use of cytokinins to induce shoot formation is well documented. Cytokinin hormonal balance necessary for the shoot differentiation process depends on the amount of endogenous hormones in the different tissues of *Passiflora* [8, 20–23].

The study observed low shoot induction frequencies for the yellow variety and complete absence of regeneration for the purple variety. Several authors have reported low or total absence of callus and adventitious shoot induction on media supplemented with BAP on various explants of *Passiflora* especially leaf discs [24–29]. However, 30% regeneration efficiency of shoots from leaf discs cut from a single parent leaf regenerating shoots and successful induction of multiple shoots from many leaf discs was reported [24]. The observed shoot induction disparities among yellow and purple passion fruit gave strong evidence of regeneration capacity of explants being strongly dependent on the genetic constitution of the cultivar [28, 30, 31]. It is probable that the growth regulator concentrations or type used was unable to stimulate cell division and organogenesis amongst the cells of the purple passion fruit leaf discs compared to yellow passion fruit leaf discs.

Similar to [24] findings, the present study also observed incidences of multiple shoot induction with a single leaf disc giving off three shoots. This was unique since most of the initiated leaf discs failed to initiate any shoot or callus. Multiple shoot induction could probably be explained by a postulation that different explants of the same genotype may respond differently to the same media composition probably due to varying gradients of endogenous hormones and physiological age of explant [32, 33].

Successful shoot induction was by both direct and indirect organogenesis. *Passiflora* regeneration via direct and indirect organogenesis on media supplemented with BAP using various explants is well documented [29, 34–36]. The study further noted that indirect organogenesis via the callus phase resulted in production of fewer plantlets although embryogenic callus induced numerous buds. The study findings were collaborated with [37] who reported that green and compact calli was the best candidate to induce shoots however the growth rate of calli was relatively difficult and took long. However, other authors expressed reservation on indirect organogenesis in preference to direct organogenesis. They argued that indirect organogenesis encouraged amitosis in the peripheral cells of calli which had the potential to result in genetic instability of the regenerated plants [35, 38].

Direct shoot induction was mainly confined to the proximal cut edges of the leaf disc pointing to the presence of embryogenic cells in this region that were most likely to dedifferentiate giving off sprouts. This morphogenic response and subsequent emergence of primordia on the injured zones is probably due to accelerated cell division reaction caused by the cut and subsequent interaction of the newly formed cells with the growth regulators in the media [25, 35].

In conclusion, several authors have successfully used leaf discs to develop transgenic passion fruit varieties [3, 39, 40]. However, a prerequisite for any successful transformation system is the availability of a regeneration system. The study was able to establish a regeneration system for Uganda’s yellow passion fruit directly from leaf discs for routine utilization with an ultimate goal of improving its agronomic value.

## Limitations

Further work needs to be done to establish why Uganda’s purple passion fruit is recalcitrant to tissue culture and also unravel the factors behind the low regeneration frequencies of the yellow variety. The study did not investigate in detail the role played by parameters like age and type of explant, cultivar genotype and plant growth regulator concentrations on regeneration efficiency.

## Additional file

**Additional file 1: Table S1.** Elongation induction of successfully regenerated yellow passion fruit shoots. **Table S2.** Effect of different NAA concentrations on root induction.

## Data Availability

Additional data has been submitted under additional file section in form of tables.

## Abbreviations

2,4-D: 2,4-dichlorophenoxyacetic acid
BAP: benzylaminopurine
GA_3_: gibberellic acid
MS Media: Murashige and Skoog medium
NAA: α-naphthaleneacetic acid
SIM: Shoot Induction Media
