# Supplementary material for: In vitro regeneration of Ugandan passion fruit cultivars from leaf discs
Source: BMC Res Notes. 2019 Jul 16;12:425. doi: 10.1186/s13104-019-4469-8 (PMC6636056; doi:10.1186/s13104-019-4469-8)
Supplement: Supplementary file 1 — Additional file 1: Table S1. Elongation induction of successfully regenerated yellow passion fruit shoots. Table S2. Effect of different NAA concentrations on root induction. [file 13104_2019_4469_MOESM1_ESM.doc]

Table S1: Elongation induction of successfully regenerated yellow passion fruit shoots

| **TREATMENT** *(n = 22;* all induced shoots were cultured onto elongation media) | | **No. of shoots initiated** | **No. of shoots that elongated** | **Average increase in height /cm** | **Shoot elongation %** | **Average**  **elongation %** |
| --- | --- | --- | --- | --- | --- | --- |
| **2.9 μM GA_3_** | Replica 1 | 5 | 1 | ˂ 0.5 | 20 | 20 |
|  | Replica 2 | - | - | - | - |  |
| **0.44 μM BAP** | Replica 1 | 5 | 5 | 3 | 100 | 95.8 |
|  | Replica 2 | 12 | 11 | 2.5 | 91.6 |  |

***Shoot elongation % =*** *[(Number of successfully elongated shoots / Total shoots initiated on elongation media) ×100]*. Total number of shoots cultured on the different elongation media was 22. Some shoots induced multiple sprout buds from the base on 0.44 μM BAP *(which increased the total number of elongated shoots for rooting).*

Table S2: Effect of different NAA concentrations on root induction

| **TREATMENT** *(n = 30;* Number of elongated shoots cultured onto RIM) | | **Number of elongated shoots initiated** | **Number of shoots that rooted** | **Average Number of roots / shoot** | **Rooting %** | **Average**  **Rooting %** |
| --- | --- | --- | --- | --- | --- | --- |
| **5.37 μM NAA** | Replica 1 | 6 | 4 | 3 | 66.6 | 72.15 |
|  | Replica 2 | 9 | 6 | 4 | 77.7 |  |
| **10.74 μM NAA** | Replica 1 | 6 | 3 | 3 | 50.0 | 47.2 |
|  | Replica 2 | 9 | 5 | 3 | 44.4 |  |

***Rooting %=****[( Number of shoots that rooted / Total shoots initiated on RIM) ×100]*. Total number of elongated shoots cultured onto the different root induction media was 30 *(observed additional shoots were induced on 0.44 μM BAP)*
